# Think Leader, Think White? Capturing and Weakening an Implicit Pro-White Leadership Bias

**DOI:** 10.1371/journal.pone.0083915

**Published:** 2014-01-08

**Authors:** Seval Gündemir, Astrid C. Homan, Carsten K. W. de Dreu, Mark van Vugt

**Affiliations:** 1 University of Amsterdam, Department of Psychology, Amsterdam, The Netherlands; 2 VU University Amsterdam, Department of Social and Organizational Psychology, Amsterdam, The Netherlands; University College London, United Kingdom

## Abstract

Across four studies, we found evidence for an implicit pro-White leadership bias that helps explain the underrepresentation of ethnic minorities in leadership positions. Both White-majority and ethnic minority participants reacted significantly faster when ethnically White names and leadership roles (e.g., manager; Study 1) or leadership traits (e.g., decisiveness; Study 2 & 3) were paired in an Implicit Association Test (IAT) rather than when ethnic minority names and leadership traits were paired. Moreover, the implicit pro-White leadership bias showed discriminant validity with the conventional implicit bias measures (Study 3). Importantly, results showed that the pro-White leadership bias can be weakened when situational cues increase the salience of a dual identity (Study 4). This, in turn, can diminish the explicit pro-White bias in promotion related decision making processes (Study 4). This research offers a new tool to measure the implicit psychological processes underlying the underrepresentation of ethnic minorities in leadership positions and proposes interventions to weaken such biases.

## Introduction

While the first African-American president in US-history is re-elected for a second term in the White House, ethnic minorities in the Western world still have difficulties finding their way to higher hierarchical positions in the labor market [1], [2]. Much like women, ethnic minorities seem to face a glass ceiling that hinders their vertical career development [3], [4]. This is problematic for several reasons. First, barriers in attaining leadership positions as perceived by members of ethnic minority groups could cause this group to psychologically disengage and devalue success in these areas [5]. Furthermore, having ethnic minority leaders gaining prominence as role models for others with similar backgrounds reduces the negative effects of stereotype threat and counters “race-based performance differences” [6], [7]. Third, ethnic minority leaders can serve as anti-stereotypical examples that reduce implicit prejudice towards minorities, at least temporarily [8]. Considering the significance of (ethnic) diversity for team and organizational performance [9], the societal need for recruiting and selecting the most qualified individuals in leadership positions, and preventing an unnecessary loss of (ethnic minority) talent, it is surprising that the number of studies on this subject is somewhat limited (for exceptions, see [10], [11]).

Across the social science disciplines, a variety of explanations have been offered for the underrepresentation of ethnic minorities in leadership positions. Sociologists have focused on how status inequality on the societal level can cause power disparities in small groups by influencing performance expectancies towards women and minorities [12]–[14]. Some industrial economics models suggest that inequalities originate from decision makers' reliance on groups' anticipated performance averages [15], and their risk aversion tendencies [16]. Social psychology offers a complementary perspective -that likely precedes these explanations- which we develop and test here. We conjecture that a major cause of the underrepresentation of ethnic minorities in leadership positions in the Western world is that this group does not fit the predominant “image” or prototype of a leader.

According to leadership categorization theory [17], an enhanced fit between a target individual's characteristics and the perceiver's implicit ideas about a typical leader (i.e., leadership prototypes) leads to positive leadership evaluations and effective leadership perceptions. This process of matching can result in: (1) the classification of the target as a (non)leader and, (2) “*a pattern-completion process through which unobserved but prototypical traits or behaviors are also associated with the categorized individual*” ([18], p. 961). We argue that a leadership bias towards ethnic minorities can occur in both of these aspects. First, people expect (business) leaders to be White, so when they estimate an individual's organizational role, they assume White targets to have leadership positions to a much larger extent than objective information (i.e., racial demographic composition of a company) would suggest [11]. Second, unobserved typical leadership traits can be mistakenly more strongly associated with individuals from some groups than others which then affects leadership evaluations.

In this paper, we show that *on the eye race neutral* (effective) leadership traits (e.g., ambitious, decisive) are more strongly associated with White-majority group members than ethnic minorities at least in Western societies. Across four studies, we uncover that leadership roles are more strongly associated–automatically and largely unconsciously–with White-majority group members than with ethnic minorities, and we expose the association between universal leadership prototypes with racio-ethnic categories (i.e., White-majority versus ethnic minority) in the Western world. Consistent with work on intergroup relations [19], [20], we argue and show that the implicit pro-White leadership bias can be (at least to some extent) suppressed through re-categorization techniques (e.g., [19], [21]).

We first introduce Leadership Categorization Theory, and discuss previous work on the pro-White leadership bias. Subsequently, we build on work on intergroup relations to highlight strategies (i.e., re-categorization) to suppress the pro-White leadership bias. The studies shed light on the possible antecedents of ethnic minorities' underrepresentation in organizational leadership positions and test ways to counter it.

## Leadership Categorization and the Pro-White Leadership Bias

Leadership Categorization Theory (LCT; [17]) applies insights from classical categorization perspectives to a leadership context. Categories are “cognitive structures that represent knowledge about a stimulus (e.g., automobiles, leaders) and its attributes” ([22], p. 21). They can help structure large amounts of information into efficient formats by “allowing us to treat many categorized stimuli as equivalent” ([22], p. 21; [17]) and to discriminate easily between information originating from different categories [23]. During this process a target's resemblance to, or divergence from the prototypes (i.e., typical characteristics of category members) guides perceivers' categorization tendencies [17], [23]. Accordingly, LCT explains perceived leadership as a result of cognitive processes during which the evaluator mentally matches the characteristics of the *target* individual with existing leader prototypes [17]. When there is a match, i.e. if the target's characteristics fit perceivers' leader prototypes, the favourability of their leadership is enhanced [24], [25]. Attributes such as intelligence, decisiveness, self-confidence, ambition, and reliability are universally considered prototypical leadership traits [26]–[31], whereas traits such as egotism, ruthlessness, and dishonesty are considered the antithesis of leadership [32].

Typical attributes of a category membership (e.g., “leader”) can be inferred from the specific exemplars of that category [29], [33] Because most of the higher hierarchical level and leadership positions in the Western world are occupied by members of the White-majority group (e.g., [2], [34]), the concomitant frequent experiences with White leaders are likely to cause perceivers to assume leaders in general to be White. Indeed, in the US, business leaders (compared to those in subordinate positions) are assumed to be White, and from this perspective a pro-White leadership bias in both perception and evaluation of leadership potential and effectiveness may have developed [11].

As perceivers evaluate a target's leadership potential and/or effectiveness, they assess the extent to which the target's attributes and/or behavior are in line with their pre-existing prototypes [29]. During this process of “matching” the target to a pre-existing prototypical image, perceivers can use partial information to infer leadership patterns, which can cause prototype consistent “false-positives” [35]. Thus, a pro-White leadership bias may involve the (mis)categorization of prototypical leadership attributes as typical for White-majority group members. Earlier research hints at this possibility. High status groups (e.g., White-majority), for instance, are seen as more competent, a construct highly related to leadership [36]. Furthermore, Livingston and Pierce [25] uncovered that having a baby face – which is considered a non-typical leader attribute [37] – is negatively correlated with success among White males in high positions of leadership, whereas it appears to have a “disarming” effect on Black CEO's.

In addition to targets' fit to universal leadership prototypes, their embodiment of group level prototypes can play a role in leadership perception and perceived effectiveness [38], [39]. That is, as targets are seen as more prototypical for the group, their leadership evaluations will be more favorable among those who identify strongly with the group [40]. From this perspective, the pro-White leadership bias should be stronger among White-majority members than ethnic minority members for two reasons. First, the White group members have a stronger association of universal leadership traits with White-majority targets. Second, White group members (compared to ethnic minority members) should have more positive leadership evaluations of White-majority targets because of their in-group prototypicality. Research on leadership (vs. subordinate) roles, however, shows that both White-majority and ethnic minority group members assume leaders in general to be White [11]. Furthermore, targets' in-group prototypicality does not necessarily affect the perceivers' evaluation of their fit to general leadership prototypes [41], which is the focus of current research. We, thus, expect to observe the implicit pro-White leadership bias in both White and non-White perceivers.

In sum, we argue that typical leadership traits are more strongly associated with White-majority group members than with ethnic minority members, and this should manifest itself in explicit evaluations of the leadership potential of the target. Although some research has questioned the (individual) level predictive value of implicit measures of explicit behavior [42], meta-analyses have shown a moderate, positive relationship between perceivers' implicit biases and their responses on related explicit measures [43]–[45]. Our first goal here is to test the emergence of an automatic, subconscious pro-White leadership bias and its effects on decisions to promote White-majority candidates into higher level leadership positions in hypothetical business settings.

## Weakening the Pro-White Leadership Bias

The second goal of our paper is to examine the effectiveness of specific cognitive strategies that can be used to weaken pro-White leadership biases. According to the LCT, perceivers' cognitive simplifications (e.g., categorization) are likely to cause perceptual biases towards minority groups. Lord and Maher's [17] discussion on gender bias in this context is relevant for the pro-White leadership bias: “*…in many instances initial exposure to a female manager results in the immediate categorization of her into a female category, as opposed to a manager or leader category. […] this type of processing may also be a source of bias.*” (p. 99). Thus, the immediate categorization of a target individual as, for instance, “Hispanic American” could inhibit his/her categorization as a “potential leader” which, in turn, would restrict his/her chances to emerge as a leader as well as being perceived as an effective leader.

One possible way to restrain this bias is to focus on the situational sensitivity of the prototype activation process. More recent views on leadership categorization reject the “rigid” aspects of information processing from the original LCT (i.e., context independent prototype activation) and embrace a connectionist model [46], [47]. According to the connectionist view of leadership categorization, information originating from various sources (individual, task-related, social context etc.) can co-act and create “contextually sensitive leadership categories or behavioral scripts” [47]. Indeed, research has shown that situational factors can determine patterns of prototype activation and perceived leader effectiveness [48], [49].

We argue that, if the pro-White leadership bias emerges from immediate categorization of a target as *ethnic minority* - which then suppresses their possible categorization as a *potential leader* - reducing this tendency can also help suppress this type of bias. Here we focused on a strategy proven to reduce intergroup differentiation (i.e., the perception of racial-ethnic groups as separate subgroups): increasing the salience of a dual identity.

### Dual Identity Suppresses the Pro-White Leadership Bias

Intergroup differentiation tends to be substantially reduced under various forms of re-categorization. The Common Ingroup Identity Model (CIIM; [19], [21]) specifies two forms of re-categorization–emphasizing an overarching, common identity, and creating a dual identity. Introducing an overarching level of identification (e.g., being European) that is inclusive of all the subgroups (e.g., being German, Dutch, French) reduces subgroup categorization and mitigates intergroup differentiation [21]. Indeed, introducing a common ingroup identity is associated with intergroup friendship and helping [19], [50], endorsement of international cooperative actions [51], and positive intergroup evaluations and compliance behavior [52].

The benefits of re-categorization in terms of one overarching identity may be offset by the individual's fundamental need to differentiate themselves from others [53]. Thus, a mere focus on an inclusive category may not satisfy the need for distinctiveness. Furthermore, seemingly overarching identities may be (implicitly) claimed by the members of the dominant group. In the United States, for instance, “being American” has been shown to be strongly associated with “being White” [54]. Increasing the salience of the “common ingroup identity” *American* could increase prejudice displayed by Caucasian Americans toward, for instance, African Americans [55]. These downsides are less prominent in CIIM's second strategy for overcoming intergroup differentiation; the creation of a *dual identity* within an overarching level of identification [19]. Highlighting both super- and subordinate levels of identification simultaneously can especially be relevant when it relates to group memberships that are central to individuals' social self and when visible attributes reflect group membership, such as racial-ethnic groups ([56]; for evidence see e.g., [57], [58]). Thus, when a dual identity is made salient, ethnic minority targets will not be perceived as such prior to the possibility to be perceived as a potential leader. Accordingly, we expect that a dual identity suppresses the pro-White leadership bias.

Taken together, if the pro-White leadership bias flows from the immediate categorization of a target as a member of a racial/ethnic group which restricts his/her perceived leadership potential, we should find that emphasizing a dual identity reduces the pro-White leadership bias. Examining this possibility is the second main goal of the current research.

## The Present Research

Our first goal - to capture the pro-White leadership bias - was pursued in the first three studies. In all three studies, we focused on implicit evaluations using standard and newly developed implicit association tests (IAT, [59]). The IAT measure is based on the assumption that stronger associations between two or more categories are cognitively more easily accessible than weaker associations, and as a result, reactions times for the earlier combination are shorter than for the latter [59], [29]. This approach enabled us to demonstrate that an implicit pro-White leadership bias exists and is present regardless of the perceiver's ethnicity (i.e., both majority and minority members should display pro-White leadership bias). To our knowledge this is the first time this specific type of bias is recorded using the IAT. Our second goal–to uncover strategies that reduce pro-White leadership bias–was pursued in Study 4. Study 4 focused on re-categorization by means of instigating a dual identity. In Study 4 we complemented the implicit measures of bias with an explicit measure of promotion-related decision making.

The studies were approved by the ethics committees from the Department of Social and Organizational Psychology at the VU University Amsterdam and Program Group Work and Organizational Psychology at the University of Amsterdam (IRB-code 2012-AO-2666), and written informed consent was obtained from the participants. No minors/children or vulnerable groups were involved as participants in the studies. Our initial *N*'s for the four studies were respectively 43, 89, 100, and 70. In each study, we lost or excluded the data of several participants because of technical errors (i.e., lost/not-recorded [SA-]IAT data), procedural errors (e.g., in Study 4 some participants accidentally wore a T-shirt in the control condition or did not wear one in one of the remaining conditions), or issues with categorizing them into one of the ethnic groups due to lack of clarity in the self-reports. For Study 4, we did not pre-select participants based on ethnicity but entered and analyzed only the data of native-Dutch participants. More information can be obtained from the first author.

## Study 1: Implicit Association between Leadership Roles and Ethnicity

The first study was a conceptual replication of Rosette and her colleagues[11]. We hypothesized that organizational leadership roles are more strongly associated with White-majority members than with ethnic minority members among both White-majority and ethnic minority participants (Hypothesis 1).

### Study 1 Method

#### Participants and Procedure

Participants were 40 students (14 men, 26 women; *M_age_* = 22.15, *SD_age_* = 3.17) at a large Dutch university. Twenty-seven participants had a native-Dutch ethnic background, and 13 had an ethnic-minority background (of which five were Surinamese-Dutch, three Asian-Dutch, two Turkish-Dutch, one Moroccan-Dutch, and two other non-native Dutch). In this and the following studies, ethnic group categorization was based on participants' self-reports and was measured after the reaction time measures. We did not control for variables such as social economic status, as an implicit pro-White leadership bias finds its nascence in a basic categorization error as a result of repeated experiences with White leaders [11] which does not necessarily differ among those at different layers of the social ladder. Participants received extra course credits or €2 for their participation. They were seated in a cubicle with a desk and a computer and completed the Ethnicity-Organizational Roles IAT on the computer. After the IAT, the participants filled out a demographics questionnaire (e.g., gender, age, ethnic background).

#### The IAT Measure

In designing the Ethnicity-Organizational Roles IAT we followed the guidelines by Greenwald and his colleagues[60]. The measure consisted of seven blocks. In Block 1 (practice, 20 trials), participants were asked to categorize native-Dutch names by pressing the key “Q”, and ethnic-minority names by pressing the key “P”. In Block 2 (practice, 20 trials), they categorized high status roles by pressing the key “Q”, and low status roles by pressing the key “P”. In Block 3 (practice, 20 trials) and Block 4 (test, 40 trials), the target and the attribute concepts were combined. The participants pressed the key “Q” if the stimulus belonged to one of the two categories “native-Dutch or high-status” and they pressed the key “P” if the stimulus was a part of “ethnic-minority or low status”. In Block 5 (reversed practice, 20 trials), the participants were asked to press “Q” if the stimulus belonged to the category “ethnic minority”, and to press “P” if the stimulus was part of “native-Dutch”. In Block 6 (reversed combined practice, 20 trials), and Block 7 (reversed combined test, 40 trials), participants pressed the key “Q” to indicate that the stimulus belonged to either “ethnic-minority” or “high status”, and pressed “P” if it belonged to either “native-Dutch” or “low status” (see Table 1 for an overview). The order in which the participants completed Block 3 (B3) and Block 4 (B4) *and* Block 6 (B6) and Block 7 (B7) was counterbalanced.

**Table 1:** Procedure of the Ethnicity-Organizational Roles IAT (Study 1).

Block	No. of Trials	Function	Items Assigned to Left Key Response	Items Assigned to Right Key Response
1	20	Practice	Native-Dutch names	Ethnic-Minority names
2	20	Practice	High status roles	Low status roles
3	20	Practice	Native-Dutch names +	Ethnic-Minority names +
			High status roles	Low status roles
4	40	Test	Native-Dutch names +	Ethnic-Minority names +
			High status roles	Low status roles
5	40	Practice	Ethnic-Minority names	Native-Dutch names
6	20	Practice	Ethnic-Minority names+	Native-Dutch names+
			High status roles	Low status roles
7	40	Test	Ethnic-Minority names+	Native-Dutch names+
			High status roles	Low status roles

*Note.* IAT =  Implicit Association Test.

Prior to the response time measure, participants were made familiar with the target-concept categories (i.e., native-Dutch and ethnic minority), the attribute categories (i.e., high status and low status organizational roles) and the stimuli. We used 20 male names (ten native-Dutch [e.g., Jasper, Alex, Onno], and ten Arab-Dutch names [e.g., Ayoub, Hamza, Bilal]) as the stimuli for the two target-concept categories (also see [61]). For the attribute category we used five high status organizational roles (i.e., boss, supervisor, leader, executive, and authority), and five low status organizational roles (i.e., helper, assistant, subordinate, aid, and follower; adapted from [62]).

### Study 1 Results and Discussion

We followed the improved scoring procedure [59] to calculate an IAT score, *D*, for each participant. We used the data from B4, B5, B6, and B7. After calculating the mean response time for correct responses per block, we replaced each incorrect response with the mean of that specific block and added a 600 millisecond “error penalty”. We computed pooled standard deviations for B3 and B6 *and* for B4 and B7. We calculated the corrected means for each of the four blocks and computed two subtractions: B6−B3 and B7−B4. We divided each outcome by the relevant pooled standard deviation. The average of the two quotients yielded an IAT score, *D*, for each participant. A positive IAT score implies a stronger association between high-status organizational roles and native-Dutch relative to the association between high-status organizational roles and ethnic-minorities, a negative score implies the reversed relationship, and a zero score implies no association between ethnicity and organizational roles.

In line with Hypothesis 1, the mean IAT scores of the participants was positive (*M* = 0.24, *SD* = 0.48, *d* = 0.50; see [63]), and differed significantly from zero *t* (39) = 3.17, *p* = .003. To investigate possible ethnicity effects we created a new dichotomous variable “participant ethnicity” (native-Dutch versus other). The mean IAT-score of the native-Dutch participants (*M* = 0.20, *SD* = 0.43), and for the ethnic-minority participants (*M* = 0.33, *SD* = 0.59), did not differ from one another *t* (38) = −0.81, *p* = .43. These results support the prediction that organizational leadership roles are more strongly associated with White-majority group members than ethnic-minority individuals (Hypothesis 1) by the members of both racial-ethnic groups.

## Study 2: Implicit Association between Leadership Traits and Ethnicity

Study 1 showed the expected automatic association between target's ethnicity and leadership roles. However, the mean IAT-score derived from the task used in Study 1 is a “relative” effect: it represents a difference in the strength of the association between the combination “White-majority  =  leadership roles, ethnic minorities  =  subordinate roles” and “White-majority  =  subordinate roles, ethnic minorities  =  leadership roles” [64]. That is, it is unclear whether the implicit bias we found is driven by a stronger association between White-majority and leadership roles or between ethnic minority and subordinate roles. In Study 2 we eliminated this limitation by relying on an SA-IAT [65] in which the categories that represent different ethnicities (i.e., native-Dutch and ethnic minority) are linked to a single attribute (i.e., leader). The use of an SA-IAT enabled us to directly test our second prediction that universal leadership traits are more strongly associated with White-majority group members than with ethnic minorities among both White-majority and ethnic minority participants (Hypothesis 2). In the SA-IAT developed here, participants were asked to categorize stimuli into one of the categories “native-Dutch” (i.e., White-majority), “ethnic minority” or the attribute “leader”. Depending on the phase, the attribute shared a response key with one of the two ethnicity categories. This enabled us to compare reaction times associated with either combination and to investigate a possible bias using the (*SD* corrected) difference scores.

### Study 2 Method

#### Participants and Procedure

Participants were 82 students (25 men, 56 women, 1 missing; *M_age_* = 20.80, *SD_age_* = 3.33) at a large Dutch university. 62 participants had a native-Dutch ethnic background, and 20 had an ethnic-minority background (of which four were Surinamese-Dutch, four Antillean-Dutch, four Moroccan-Dutch, three Asian-Dutch, and five other non-native Dutch). They received extra course credits or €2 for their participation. Participants were seated in a cubicle with a desk and a computer and completed the Ethnicity-Leadership SA-IAT on the computer. After the SA-IAT, the participants filled out a demographics questionnaire (e.g., gender, age, ethnic background).

#### The SA-IAT Measure

We developed a Single Attribute Implicit Association Test (SA-IAT; [60], [65]). The SA-IAT was adapted from the method proposed by Greenwald et al. [60], [65] and is similar to the Single-Target IAT [64]. The measure consisted of three blocks. In Block 1 (practice, 20 trials), participants were asked to categorize native-Dutch names by pressing the key “Q”, and ethnic-minority names by pressing the key “P”. In Block 2 (test, 35 trials), participants pressed the key “Q” to indicate that the stimulus belonged to either “native-Dutch” or “leader”, and pressed “P” if it belonged to “ethnic minority”. In Block 3 (test, 35 trials), participants pressed the key “Q” if the stimulus belonged to “native-Dutch” and pressed “P” if it belonged to either “ethnic minority” or “leader” (see Table 2 for an overview). The test blocks had a key-distribution of 2∶2∶3 (or 3∶2∶2) in order to avoid a response bias potentially caused by equal frequencies of correct responses per key [66]. The order of the two test blocks was counterbalanced.

**Table 2:** Procedure of the Ethnicity-Leadership SA-IAT.

Block	No. of Trials	Function	Items Assigned to Left Key Response	Items Assigned to Right Key Response
1	20	Practice	Native-Dutch names	Ethnic-Minority names
2	35	Test	Native-Dutch names +	Ethnic-Minority names
			Leadership traits	
3	35	Test	Native-Dutch names	Ethnic-Minority names +
				Leadership traits

*Note.* SA-IAT =  Single Attribute Implicit Association Test. In Study 4 “names” are replaced with pictures.

Prior to the response time measure, participants were made familiar with the categories (i.e., native-Dutch and ethnic minority), the attribute (i.e., Leader) and the stimuli. The stimuli used for the two categories were 10 male names (five native-Dutch [e.g., Jasper], and five Arab-Dutch [e.g., Jafaar]; also see [61]). For the attribute “Leader” we used five traits prototypically associated with leaders: Decisive, Intelligent, Self-confident, Ambitious, and Reliable [26]–[28], [31].

### Study 2 Results and Discussion

As in Study 1, we used the improved scoring procedure [53] to calculate an SA-IAT score, *D*, for each participant. As predicted in Hypothesis 2, the mean SA-IAT scores of the participants was positive (*M* = 0.27, *SD* = 0.43, *d* = 0.62), and differed significantly from zero *t* (81) = 5.64, *p*<.001. The mean SA-IAT score of the native-Dutch participants (*M* = 0.30, *SD* = 0.43), and the ethnic-minority participants (*M* = 0.16, *SD* = 0.44) did not differ from one another, *t* (80) = 1.28, *p* = .20. These results support the hypothesis that leadership traits are more strongly associated with White-majority group members than ethnic-minority individuals (Hypothesis 2), by the members of both racial-ethnic groups.

One possible limitation of this study may be that all leadership traits used as stimuli (e.g., intelligent, decisive) can be considered positive traits. Thus, the pro-White leadership bias measured in Study 2, can be an expression of a general implicit pro-White bias (i.e., an automatic association of positive words with White-majority targets). Yet, we have strong reasons to believe that our SA-IAT measure uncovers a different type of association than a mere general pro-White bias. In this respect, the results revealed that –similar to White-majority participants– ethnic minority participants showed a pro-White leadership bias. This is in contrast with previous empirical research on generalized implicit measures that reported no significant bias by ethnic minorities (i.e., IAT scores for the automatic association between positive words and target racial-ethnic groups did not differ from zero; [67], [68]). A bias in the race-ethnicity related assumptions about individuals in leadership positions (as opposed to subordinates), however, has been recorded for both White-majority and ethnic-minority perceivers [11]. Nevertheless, because the ethnicity-leadership SA-IAT is a newly developed instrument, we conducted an additional study in a different Dutch university to replicate the findings from Study 2 and to show the dissimilarities between this measure and generalized implicit prejudice measures.

## Study 3: Pro-White Leadership Bias  =  General Pro-White Bias?

Although Study 2 revealed a clear implicit pro-White leadership bias, one additional step may be useful to assure the robustness of the bias. Our goal in Study 3 was to replicate the findings from Study 2, as well as, to give a conclusive answer about its divergence from the more conventional, general implicit association measures. In this study, the participants completed both an ethnicity-leadership SA-IAT and an SA-IAT that measured a generalized implicit prejudice. We expected both White-majority and ethnic minority participants to display an implicit pro-White leadership bias (Hypothesis 3a), but only the first group to show a generalized implicit pro-White bias (Hypothesis 3b).

### Study 3 Method

#### Participants and Procedure

Participants were 94 students (18 men, 71 women, 5 missing; *M_age_* = 21.51, *SD_age_* = 3.05) at a large Dutch university. Eightyparticipants had a native-Dutch ethnic background, and 14 had an ethnic-minority background (of which eight were Turkish-Dutch, two Asian-Dutch, and four other non-native Dutch). They received extra course credits or €2,50 for their participation. Participants were seated in a cubicle with a desk and a computer and completed both an Implicit Prejudice SA-IAT and an Ethnicity-Leadership SA-IAT on the computer. Depending on the condition they either started with the Implicit Prejudice SA-IAT or the Ethnicity-Leadership SA-IAT. After the SA-IAT's, the participants filled out a demographics questionnaire (e.g., gender, age, ethnic background).

#### The SA-IAT measures

The Leadership-Ethnicity SA-IAT was identical to the instrument used in Study 2. The Implicit Prejudice SA-IAT replaced the attribute “Leader” with “Good”. Following Wigboldus, Holland, and Van Knippenberg [69], the stimuli used for the Implicit Prejudice SA-IAT were love, peace, joy, happiness, and flower.

### Study 3 Results and Discussion

In line with Hypothesis 3a, two separate one-sample t-tests revealed that both native-Dutch (*M* = 0.25, *SD* = 0.50, *d* = 0.51; *t* [79] = 4.52, *p*<.001, and ethnic minority participants (*M* = 0.23, *SD* = 0.49, *d* = 0.48; *t* [13] = 1.78, *p* = .049 [one-tailed]) showed a stronger association between leadership traits and native-Dutch than these traits and ethnic-minorities. However, only native-Dutch participants (*M* = 0.30, *SD* = 0.48, *d* = 0.61; *t* [79] = 5.48, *p*<.001) associated non-leadership related positive words more strongly with native-Dutch than ethnic minorities. The mean Implicit Prejudice SA-IAT score of the ethnic-minority participants (*M* = 0.06, *SD* = 0.51, *d* = 0.12) did not statistically differ from zero, *t* (13) = 0.46, *p* = .66 (Hypothesis 3b). We also calculated the correlation between the Implicit Prejudice SA-IAT and the Leadership-Ethnicity SA-IAT. As we would have expected, the Pearson correlation between the two measures was statistically not significant, *r* (92) = .17, *p* = .10. These findings support our assumption that the Leadership-Ethnicity SA-IAT differs from the Generalized Implicit Prejudice measures.

Studies 2 and 3 revealed (i) the presence of an implicit pro-White leadership bias that (ii) differs from the more conventional, generalized implicit prejudice measure. The ethnicity-leadership SA-IAT can, thus, be used to measure a specific implicit bias that is likely to put up to the underrepresentation of ethnic minorities in leadership positions. In Study 4 we targeted contextual factors–re-categorization–that was hypothesized to mitigate the pro-White leadership bias. In this final study we engaged White-majority group members, because this group still dominates the positions of power in organizations (e.g., [1]), and thus it is essential to uncover under which circumstances bias by the members of this group diminishes.

## Study 4: Dual Identity to Suppress the Implicit Pro-White Leadership Bias

In this study we examined dual identity (as a re-categorization strategy) as a boundary condition on the pro-White leadership bias. The target stimuli belonging to the same ethnic category were all featured in T-shirts of the same color (e.g., all native-Dutch stimuli wore blue T-shirts). Prior to the SA-IAT measure we asked the participants to wear a T-shirt in the same color as their ingroup (i.e., intergroup differentiation condition: salient ethnic groups in the absence of an overarching identity) or their outgroup (i.e., dual identity condition: salient ethnic groups within an overarching identity). We expected the participants in the intergroup differentiation and the control condition to show higher levels of a pro-White leadership bias than those in the dual-identity condition (Hypothesis 4a). Furthermore we expected that the mean SA-IAT score would be positive and significantly deviating from zero in the intergroup differentiation and control conditions, while the mean SA-IAT score would not be significantly differing from zero in the dual-identity condition (Hypothesis 4b).

The second goal of Study 4 was to investigate whether the SA-IAT scores predict discriminatory behavior during promotion related decision making processes. Earlier research has stressed biases in leadership categorization specifically as a pro-White rather than an anti-minority bias [11]. The ethnicity-leadership SA-IAT fits this idea because the SA-IAT scores reflect an *SD* corrected difference score between an implicit “pro-White” and “pro ethnic-minority” leadership bias [59]. Larger SA-IAT scores thus reflect the extent to which participants perceive a White-majority target to better fit a leadership position than an ethnic minority target. This implicit bias could influence their explicit leadership-related decision making processes, so that a higher SA-IAT score is associated with an enhanced willingness to promote a White (and not a non-White) target to a higher leadership position (Hypothesis 4c).

### Study 4 Method

#### Participants and Procedure

Participants were 67 native-Dutch students (20 males, 44 females, 3 missing; *M_age_* = 22.17, *SD_age_* = 7.57) at a large Dutch university. Participants were seated in a cubicle with a desk and a computer and completed the Ethnicity-Leadership SA-IAT on the computer. Prior to the SA-IAT measure they were either asked to wear a T-shirt (in blue or green) or they were not asked this ( = control). They were randomly assigned to either the dual identity, intergroup differentiation or the control condition.

After the SA-IAT's, the participants were asked to read the short resume of a candidate for a higher leadership position within a fictitious company. The candidate in the scenario had a lower level management position in the company as the head of a department and applied for a higher level position to become the head of the division. The scenario was accompanied by an organizational structure scheme that showed the promotion aspects of this application. The resumes were kept constant except the name of the candidate which was either a native-Dutch or an ethnic minority (in this case a Moroccan/Arab) name (see Appendix S1 for the resume). The participants were asked to imagine they were a member of the committee that made the promotion decision and rated their willingness to hire the candidate for the higher leadership position. The participants, then, filled out a demographics questionnaire (e.g., gender, age, ethnic background).

#### SA-IAT measure

The procedure of the Ethnicity-Leadership SA-IAT was identical to the studies above. The SA-IAT stimuli in this study consisted of five pictures per category (i.e., five native-Dutch and five Moroccan pictures [70]). In order to keep interethnic boundaries salient, the stimuli of the same ethnic group featured in T-shirts all in the same color. The specific T-shirt color per target ethnic group was counterbalanced. Participants were randomly assigned to one of the three conditions: (1) Participants wore the same colored T-shirt as their ethnic in-group targets (e.g., participant blue - native-Dutch stimuli blue; the intergroup differentiation condition). (2) Participants wore the same colored T-shirt as their ethnic out-group targets (the dual identity condition). (3) Participants were not given a T-shirt before the measure; all pictured individuals wore black T-shirts ( = control).

#### Promotion

The participants' willingness to promote the candidate to a higher leadership position was measured using one item: “I would hire this candidate for the position of the head of the division.” The participants rated on a 7-point Likert scale (1 =  completely disagree, 7 =  completely agree) to what extent they agreed with the statement.

### Study 4 Results and Discussion

#### SA-IAT effects per condition

An SA-IAT score, *D*, per participant was calculated using the same algorithm as before. A one-way analysis of variance (ANOVA) showed that the conditions differed from one-another, *F*(2, 64) = 3.78, *p* = .028, ηp^2^ = .11. Contrast analyses comparing the dual identity condition to the remaining two conditions partially supported Hypothesis 4a, showing that the mean SA-IAT score in the dual-identity condition (*M* = 0.08, *SD* = 0.44, *d* = 0.19) was lower than the control condition (*M* = 0.38, *SD* = 0.49, *d* = 0.77), *t*(64) = −2.34, *p* = .023, but not different from the inter-group differentiation condition (*M* = 0.10, *SD* = 0.26, *d* = 0.40), *t*(64) = 0.15 *p* = .88. Furthermore, results revealed that the participants in the intergroup differentiation condition (*t* [27] = 2.09, *p* = .046), and in the control condition (*t* [20] = 3.54, *p* = .002) showed significantly stronger associations between native-Dutch and leadership traits than between ethnic minority and leadership traits (i.e., their scores significantly differed from zero). However, scores of participants in the dual-identity condition did not differ from zero (*t* [17] = 0.80, *p* = .44), indicating bias suppression is this condition. Supporting Hypothesis 4b, these results suggest that introducing a dual identity can weaken the implicit pro-White leadership bias.

#### Promotion

To investigate whether the implicit bias assessed by the ethnicity-leadership SA-IAT can predict intentions to hire a majority (and not an ethnic minority) candidate for a higher leadership position, we conducted a multiple regression analysis. After centering the SA-IAT scores, and creating dummy variables for the candidates' racial background (White = 0, Minority  = 1), we computed the interaction term for SA-IAT score-by-candidates' racial background [71]. We entered these variables simultaneously as independent variables in a regression analysis and identified “willingness to promote” as the dependent variable. Higher SA-IAT scores predicted participants' increased intentions of promoting a candidate to a higher leadership position, *B* = 1.34, *SE* = .49, *t*(62) = 2.76, *p* = .008, while candidates' racial background did not have a significant main effect on the participants' intentions to promote them to a higher leadership position, *B* = 0.11, *SE* = .25, *t*(62) = 0.44, *p* = .66. Importantly, there was a significant two way interaction between SA-IAT scores and candidates' racial background, *B* = −1.29, *SE* = .62, *t*(62) = −2.09, *p* = .041. Simple slopes for White and ethnic minority candidates were tested for high (+1 SD) and low (−1 SD) levels of SA-IAT scores (Aiken & West, 2001). As expected higher levels of the SA-IAT effect predicted willingness to promote a White candidate to a higher leadership role, *B* = 1.34, *SE* = .49, *t*(62) = 2.76, *p* = .008, while this effect did not predict the participants' willingness to promote an ethnic minority candidate to a higher leadership position, *B* = 0.04, *SE* = .39, *t*(62) = 0.11, *p* = .91. Thus, supporting Hypothesis 4c, a larger score on the ethnicity-leadership SA-IAT is associated with positive intentions to hire a White-majority candidate into a higher leadership position, and is unrelated to the intention to hire an ethnic minority candidate.

Study 4 showed that introducing a dual identity (i.e., having an overarching level of categorization while keeping racial-ethnic boundaries salient) can help suppress the implicit pro-White leadership bias. Moreover, the results also showed that this bias can influence promotion related decision making processes. These results support the view that re-categorization (e.g., [19]) can be an effective strategy to repress the pro-White leadership bias. This finding is especially relevant, because this bias can restrict the higher level leadership possibilities of ethnic minorities by causing discriminatory behavior during promotion-related decision making processes.

Although the bias for the intergroup differentiation and the control conditions were both statistically significant, it is surprising that the mean SA-IAT scores in the former were clearly lower than the latter. Moreover, although we did not predict this, the intergroup differentiation condition was significantly lower than the control condition. This finding does not fit the theory as this condition arguably amplifies subgroup categorization and thus should have been at least as high as (or higher than) the control condition. One possible explanation for this effect is that wearing a T-shirt in itself might have created an overarching level of identification which also reduced the mean bias in the intergroup differentiation condition. Because both ethnic target groups were featured in T-shirts in striking colors, the intended amplification of differences between the ethnic categories may have become less salient. Another possible explanation – as pointed out by an anonymous reviewer- is that by making intergroup boundaries overly salient, this condition might have activated bias control, and as a result might have depressed the SA-IAT effect altogether. Nevertheless, the participants in the intergroup differentiation condition still expressed a significant level of pro-White leadership bias (and those in the dual identity condition did not) which tells us that the intergroup differences were –at least to some extent- processed.

## General Discussion

Across four studies, we integrated leadership categorization theory (e.g., [17]) and theory on intergroup relations (e.g., [19]) to investigate the possible intra-individual antecedents of the underrepresentation of ethnic minorities in leadership positions as well as offered contextual interventions to restrict this. Earlier research suggested that frequent experiences with White-leaders have created a pro-White leadership bias in the Western world, which is –to some extent- responsible for the underrepresentation of racial-ethnic minorities in leadership positions [11]. In this research we focused on an *implicit* pro-White leadership bias. In line with the hypotheses, the results showed that organizational leadership roles (Study 1), and more importantly, universal leadership traits are more strongly associated with White-majority group members than ethnic minorities (Study 2 & 3). Crucially, this bias can be weakened by increasing dual levels of identification (i.e., recategorization; Study 4), which can suppress explicit pro-White biases during promotion related decision making processes (Study 4).

Our findings extend previous research [11] by showing that the association between leadership roles and targets' ethnic background is -at least partially- an implicit, non-deliberative process which also occurs in a European context. The studies also provide insight into bottom-up aspects of the pro-White bias in leadership categorization by showing the association between universal leadership prototypes with racial-ethnic categories [18]. Previous research has demonstrated similar implicit bias for sex [72], but not for ethnicity. Although the existence of such a bias may be discouraging, the possibility to “turn it off” by using rather easy-to-apply cognitive interventions, which in turn can diminish explicit pro-White bias in promotion-related staffing decisions, is promising.

Our findings advance theory by assessing novel hypotheses about racial-ethnic biases in leadership categorization and the conditions under which these biases can be weakened. This research answers the call for investigating the intersecting areas of diversity and leadership research [73] by showing that the perspectives from either of these areas can make meaningful contributions to the other. Specifically, supporting the connectionist view [47], our results clearly demonstrate that racial-ethnic biases that may occur during the prototype activation process are sensitive to situational cues. Applying strategies from intergroup relations perspectives can be helpful in restricting this type of leadership bias. Our findings likely translate to leadership categorization bias towards other leadership minorities such as women and sexual minorities as they combat bias in its nascence. Furthermore, these results extend and complement research on leadership emergence [74] and perceivers' evaluations of counter-stereotypical leaders under varying conditions and situational cues [75], [76].

This research provides instruments for work organizations to restrict pro-White leadership bias and accommodate ethnic minority employees in their vertical career development. The leadership-ethnicity SA-IAT is an easily applicable assessment and selection tool to determine decision makers' level of bias. The results of the measure can be used as an instrument to increase awareness of the implicit bias towards ethnic minority leadership. When explained thoroughly, this measure can be a valuable tool in diversity training practice and inform the trainees on ways to combat the behavioural consequences of this type of bias [77]. Furthermore, our results show that the implicit pro-White leadership bias may be weakened when multi-group memberships are made salient (Study 4). Organizations can, for instance, focus on diversity policies that recognize and value diverse characteristics of its employees [78], [79].

Although our work specifically focuses on implicit, automatic processes, we cannot rule out that more explicit, deliberative processes may play a role in these responses. For instance, some research shows that prototype-consistency plays an important role in how White versus African-American leaders and subordinates are explicitly evaluated [80]. While prototype-consistent White leaders are evaluated (e.g., achievement motivation) more positively than White subordinates, African-American leaders are evaluated less positively than African-American subordinates [80]. These results suggest that, because changes to status quo may be threatening for the members of the White-majority group, who on average have a disproportionably large access to scarce resources (e.g., leadership positions), they may be both consciously as well as unconsciously motivated to act in ways that retain social arrangements as they are [80]. Moreover, these biases may both consciously and unconsciously help retain and justify the status-quo, which in turn might protect both those who are advantaged (e.g., White-majority) and disadvantaged (e.g., racial-ethnic minority) by the status-quo from negative psychological consequences (e.g., negative affect; see [81]). Future research should focus on the additive, or possibly multiplicative, effects of implicit and explicit leadership biases towards different racial-ethnic groups, and the possible system-justifying motivations that may affect their strength.

Our studies have several limitations. Although controlling for the participants' gender does not influence our results, the stimuli used in these studies were male targets only; thus, we do not know if our findings generalize to female targets. Some studies suggests that this type of bias may be stronger in case of female minority targets (e.g., [82]). Recent research, however, argues that the bias is especially present towards male targets [83]. Future studies should include the targets with both genders. One possible strategy is to compare the bias strength for all possible combinations of gender and ethnicity (e.g., White female-minority male, White male-minority female).

Second, all four studies were conducted in a lab setting with university students as participants. This begs the question of whether perceivers with more work and life experience also would show a bias in Ethnicity-Leadership SA-IAT. Recent research, however, shows that even participants with considerable work experience and knowledge about stereotypes are not immune for comparable biases [84]. Yet, it is interesting and valuable to conduct similar research in work organizations and real-life promotion contexts. Furthermore, the pro-White bias found in our studies may be restricted to the Western world where Whites are the numeric majority as well as the dominant group in terms of power, and resources. How would these results translate into other parts of the world? Cross–cultural research is needed to examine the transferability of this bias to the other parts of the world.

One of the striking findings of the present research is that both ethnic minorities and the members of the White-majority group show a pro-White leadership bias in the categorization of universally valued leadership traits. Interestingly, minorities' level of bias shows greater variability across the studies than the majorities' level of bias. This may be a result of larger heterogeneity within this group than the majority group. For instance, the number of years these individuals reside in The Netherlands may have influenced their implicit leadership prototypes and thus affected their scores. Current studies cannot disentangle these aspects. However, combining the ethnic minority data from the second and the third studies (*n* = 34) shows that on average this group shows a significant implicit pro-White leadership bias (*M* = 0.19, *SD* = 0.45, *d* = 0.42; *t* [33] = 2.45, *p* = .020). Still, it may be interesting for future studies to focus on possible variables that affect minorities' pro-White leadership bias.

From an in-group prototypes view on leadership [38], [39], [85], one might argue that ethnic minority participants' scores should have shown a pro-minority rather than a pro-White leadership bias. Although our findings do not support this, it is important to note that the pro-White and pro-ingroup biases in leadership are not necessarily mutually exclusive. First, the source of the bias in both cases is the immediate categorization of the target as a member of a dichotomy (i.e., White versus minority) other than the dichotomy central to leadership evaluations (i.e., leader versus non-leader; [17]). Both types of bias, then, arguably occur as a result of categorizing a target individual as a *non-member* of the target category. Thus suppressing the perceivers' “spontaneous” categorization of the target as a member of an ethnic minority group, helps reducing the bias in leadership categorization. This explains how a bias reduction strategy derived from social and self-categorization perspectives (i.e., re-categorization; [19]) is effective in suppressing the pro-White leadership bias. Second, leadership categorization is a hierarchical model, thus, the specificity of prototypes varies between the different levels [17]. The current research focuses on the highest, most abstract level where the perceivers distinguish *leaders* from *non-leaders*. In-group prototypes may be more visible and relevant in the lower levels of the hierarchy where perceivers, for instance, distinguish between *political* and *military* leaders. Future studies should focus on the interaction between general leadership schemata [17], and within-group leadership prototypes (e.g., [39]) as a function of the different hierarchical levels of categorization.

Another important direction for future research is to examine the consequences of the pro-White leadership bias for minorities themselves. This is in line with recent perspectives that call for including stigmatized individuals in research as actors, instead of mere targets [86]. How does this bias relate to career decisions of ethnic minorities? Are minorities with high levels of bias on the ethnicity-leadership SA-IAT, for instance, less likely to pursue promotion, or to apply for leadership positions? Earlier research has, for instance, shown that stigmatized individuals' self-endorsement of stereotypes affects their perception of and performance in stereotype relevant areas [87]. It may, thus, mean that the larger ethnic-minority participants' scores on the leadership-ethnicity SA-IAT, the smaller their willingness to apply for leadership positions or to aspire organizational leadership positions. Future studies should examine possible self-selection tendencies by ethnic minorities as a function of their internalization of the pro-White leadership bias.

In sum, the general finding that people tend to associate universally valued leadership traits more with White rather than with ethnic minority categories –at least partially- illuminates why ethnic minorities may be less likely to obtain higher level leadership positions in Western society. Yet importantly, we show that relatively simple re-categorization procedures can help weaken this bias. This may limit an explicit pro-White bias in promotion decisions, which has important implications for leadership practice.

## Supporting Information

Appendix S1 Curriculum Vitae (translated from Dutch).
(DOCX)
