# Supplementary material for: Think Leader, Think White? Capturing and Weakening an Implicit Pro-White Leadership Bias
Source: PLoS One. 2014 Jan 8;9(1):e83915. doi: 10.1371/journal.pone.0083915 (PMC3885528; doi:10.1371/journal.pone.0083915)
Supplement: Appendix S1 — Curriculum Vitae (translated from Dutch). (DOCX) [file pone.0083915.s001.docx]

**Appendix A: Curriculum Vitae (translated from Dutch)**

**Personal information**

**First name:** Maarten / Mounier

**Last name:** van Baaden / el Baddoui

**Gender:** Male

**Address:** Heerenstraat 305

**Zip code:** 2563 VX

**City:** The Hague

**Telephone number:** 070-3568952

**Mobile number:** 06-44134458

**Date of birth:**  13-05-1978

**Driver’s license:** B

**Marital status:** Married

**Education**

2000 - 2004 Leiden University

Major: Communication science
Graduated in 2004

1996 - 2000 The Hague University College
Major: Communication
Graduated in 2000

**Work experience**

2008-present Company name: LEXOS B.V.

Position: Head of the marketing department

Description: management position

2004 - 2008 Company name: Terra Communications Rotterdam

Position: Communication counselor

Description: employee public relations office

2002 - 2004 Company name: KPN
Position: Helpdesk employee
Description: Helping clients with questions

**Competences**

- Creative
- Solution oriented
- High societal responsibility

**Software knowledge**

-Adobe package: Excellent
-Microsoft Office: Excellent
